# Disrupted default mode network dynamics in recuperative patients of herpes zoster pain

**DOI:** 10.1111/cns.13433

**Published:** 2020-07-16

**Authors:** Ying Wu, Chao Wang, Wei Qian, Lina Yu, Xiufang Xing, Lieju Wang, Na Sun, Minming Zhang, Min Yan

**Affiliations:** ^1^ Department of Anesthesiology the Second Affiliated Hospital Zhejiang University School of Medicine Hangzhou China; ^2^ Department of Radiology The Second Affiliated Hospital Zhejiang University School of Medicine Hangzhou China

**Keywords:** brain network, default mode network, herpes zoster, recuperation, resting‐state functional MRI

## Abstract

**Introduction:**

Previous studies of herpes zoster (HZ) have focused on acute patient manifestations and the most common sequela, postherpetic neuralgia (PHN), both serving to disrupt brain dynamics. Although the majority of such patients gradually recover, without lingering severe pain, little is known about life situations of those who recuperate or the brain dynamics. Our goal was to determine whether default mode network (DMN) dynamics of the recuperative population normalize to the level of healthy individuals.

**Methods:**

For this purpose, we conducted resting‐state functional magnetic resonance imaging (fMRI) studies in 30 patients recuperating from HZ (RHZ group) and 30 healthy controls (HC group). Independent component analysis (ICA) was initially undertaken in both groups to extract DMN components. DMN spatial maps and within‐DMN functional connectivity were then compared by group and then correlated with clinical variables.

**Results:**

Relative to controls, DMN spatial maps of recuperating patients showed higher connectivity in middle frontal gyrus (MFG), right/left medial temporal regions of cortex (RMTC/LMTC), right parietal lobe, and parahippocampal gyrus. The RHZ (vs HC) group also demonstrated significant augmentation of within‐DMN connectivity, including that of LMTC‐MFG and LMTC‐posterior cingulate cortex (PCC). Furthermore, the intensity of LMTC‐MFG connectivity correlated significantly with scoring of pain‐induced emotions and life quality.

**Conclusion:**

Findings of this preliminary study indicate that a disrupted dissociative pattern of DMN persists in patients recuperating from HZ, relative to healthy controls. We have thus provisionally established the brain mechanisms accounting for major outcomes of HZ, offering heuristic cues for future research on HZ transition states.

## Graphical abstract

It is the first time to demonstrate that a disrupted dissociation pattern of DMN still exists in recuperative herpes zoster patients compared with healthy controls even the rash disappeared and pain was relieved. This study could provide heuristic cues for future research about herpes zoster transition mechanism.

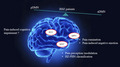

## 1 INTRODUCTION

Herpes zoster (HZ), commonly known as shingles, represents a recrudescence of latent varicella‐zoster virus (VZV) that inflames and injures spinal or cranial sensory ganglia. Typically, a unilateral erythematous rash develops in affected skin, accompanied by various pain sensations, such as burning, stabbing, soreness, bloating, or allodynia.^1^, ^2^ One epidemiologic study has determined that ~12.5% of older patients (≥50 years) with HZ may experience postherpetic neuralgia (PHN) 3 months after onset of zoster, and the proportion rises with advancing age.^3^ As the most common and intractable sequela of HZ, PHN has received the most attention to date, given its effects on patient quality of life and the considerable social burden inflicted.^4^, ^5^ To understand its mechanism for precision treatment, a number of neuroimaging changes have been identified in patients with acute HZ or PHN, compared with healthy individuals, particularly exhibiting abnormal function in classic sensory‐discriminative and emotional‐linked areas (ie, thalamus, insula, frontal lobe, brainstem, temporal lobe, and limbic lobe).^6^, ^7^, ^8^, ^9^, ^10^

However, after comparable episodes of acute shingles, the majority of patients gradually recover, and the pain attenuates. Lack of prescribed treatments or clinical follow‐up in this recuperative population has hampered investigations, creating a scarcity of data on their life situations and brain dynamics. Procuring such data is indispensable in deciphering HZ transition mechanism. The present preliminary study was conducted to ascertain whether brain dynamics in patients recuperating from HZ normalize to the level of healthy individuals.

In previous efforts, widely distributed abnormalities of brain activity in acute and chronic pain have reflected multidimensional dysfunction of pain sensation, pain‐related emotion, and cognition.^11^ An isolated, predefined brain area is simply incapable of such complexity. A better understanding of pain‐related neurobiology would thus require investigations of brain networks. Resting‐state networks (RSNs) have proven important in high‐order brain functions and neuropsychiatric disorders,^12^, ^13^, ^14^ incorporating brain regions with coherent neuronal activity of low‐frequency blood oxygenation level‐dependent (BOLD) signal fluctuations.^15^ Among the various known RSNs, the default mode network (DMN) is seemingly the most widely studied and well characterized, supporting internal mentation (such as memory, prospection) and acting as a sentinel to monitor the external environment.^16^, ^17^, ^18^, ^19^, ^20^ Interest has been considerable in terms of correlating the DMN with pain intensity, negative mood, or pain rumination.^21^, ^22^, ^23^ Recent investigations have reported disrupted DMN dynamics in patients with multiple acute and chronic pain conditions, including chronic low back pain and fibromyalgia.^21^, ^23^, ^24^, ^25^, ^26^, ^27^ These studies suggest that both acute and chronic pain may reorganize DMN dynamics to reflect the undesirable physiology of differing pain conditions.

It is worth noting that acute HZ and PHN (vs healthy controls) similarly disrupt activity of DMN nodes (such as medial prefrontal cortex [mPFC], precuneus [PCu], left medial/lateral temporal cortex [MTC/LTC], and frontal lobe).^6^, ^7^, ^8^, ^10^, ^28^ Our present study was focused on recuperative states of HZ, and the primary aims were as follows: (a) use of independent component analysis (ICA), a robust technique based on blind source separation, to determine whether DMN dynamics and the intrinsic functional connectivity (FC) thereof would remain disrupted in patients recuperating from HZ, relative to healthy controls (HCs); and (b) if true, whether clinical symptom‐associated measures are related to these FC changes. Our findings might then pinpoint brain mechanisms accounting for major recuperating outcomes of HZ, providing heuristic cues for future research on HZ transition states.

## 2 METHODS

### 2.1 Selection of study subjects

All study procedures described herein were approved by the Ethics Committee of the Second Affiliated Hospital of Zhejiang University School of Medicine. Each enrollee granted written informed participatory consent. Overall, 30 right‐handed patients recuperating from HZ (RHZ group: mean age ± standard deviation [SD], 61.63 ± 6.32 years) and 30 age‐matched right‐handed healthy controls (HC group: mean age, 59.07 ± 7.47 years) were recruited for study. The PHN diagnostic criteria are often defined by the International Association for the Study of Pain (IASP).^29^ In contrast to the PHN, our recuperating patients were diagnosed by a clinician, using the following criteria: (a) self‐appraised pain intensity score <4/10 by visual analog scale (VAS: 0 [no pain] to 10 [worst pain imaginable]); (b) ≥3‐month duration after onset of acute shingles; and (c) no medical treatment rendered for HZ. Exclusion criteria for both RHZ and HC groups were the following: (a) special HZ (of ear, eye, or viscera or asymptomatic); (b) history of ongoing acute or chronic pain attributable to headaches, toothaches, arthritis, or cervical/lumbar spondylopathy; (c) psychiatric or neurological disorders (eg, epilepsy or head injury); (d) any severe major health condition, such as cardiovascular disease or renal insufficiency; and (e) contraindications to MRI.

### 2.2 Assessment of pain and emotional parameters

All questionnaires were completed 1 hour prior to brain scans. Each participant completed the McGill pain questionnaire (MPQ) short form, the chief elements being 12 sensory and 4 affective descriptors. VAS, present pain intensity (PPI), and ID Pain scores for assessing neuropathic pain were also completed by each. Depression and anxiety states were assessed via Hamilton Depression (HAMD) and Hamilton Anxiety (HAMA) Scales. Participants also completed the two‐part Positive Affect Negative Affect Schedule (PANAS) and the Medical Outcomes Study (MOS) 36‐item short‐form survey (SF‐36) on health‐related life quality.

### 2.3 Acquisition of fMRI data

All MRI scans were performed using a 3.0‐Tesla MRI scanner (GE Discovery 750; GE Healthcare) equipped with an 8‐channel head coil. During these procedures, each patient was positioned supine, the head firmly restrained by foam pads; and earplugs were provided to reduce noise during scanning. They were asked to remain still as long as possible, keeping eyes closed but staying awake. High‐resolution structural T1‐weighted images were acquired using a fast spoiled gradient recalled echo pulse sequence as follows: repetition time (TR) = 7.3 ms; echo time (TE) = 3.0 ms; field of view (FOV) = 260 × 260 mm^2^; flip angle = 11°; matrix size = 256 × 256; slice thickness = 1.2 mm; and 196 continuous sagittal slices. Resting‐state fMRI images were obtained using a gradient recalled echo (GRE)‐echo planar imaging (EPI) sequence: TR = 2000 ms, TE = 30 ms, flip angle = 77^°^, FOV = 240 × 240 mm^2^, matrix = 64 × 64, slice thickness = 4 mm, slice gap = 0 mm; and 38 interleaved axial slices.

### 2.4 Resting‐state fMRI (rs‐fMRI) preprocessing

In preprocessing of fMRI time series volume data, the Resting‐State fMRI Data Analysis Toolkit (REST, V1.8; http://www.restfmri.net) and Statistical Parametric Mapping (SPM12; http://www.fil.ion.ucl.ac.uk/spm) were accessed, using MATLAB platform (MathWorks). The first 10 volumes of each functional time series were discarded to avoid transient signal changes before magnetic field steady states were reached, allowing subjects to acclimate in this scanning environment. We then corrected the other images for timing differences (slice 37 used as reference) and head motion, determining translation (mm) and rotation (degrees) by six parameters (three each, translation and rotation). There were no group‐wise exclusions due to head motion beyond 2 mm of displacement or 2° of rotation. Subsequently, we spatially normalized images to the Montreal Neurological Institute (MNI) space using EPI templates with resampling voxel sizes of 3 × 3 × 3 mm. All images generated were spatially smoothed using a Gaussian kernel of 6 × 6 × 6 mm, full width at half maximum (FWHM).

### 2.5 Identification of DMN via Independent Component Analysis (ICA)

Independent component analysis is a powerful tool based on data‐driven blind source separation that captures essential components of multivariate rs‐fMRI signals, extracting independent sources from mixed sources.^30^, ^31^ Concatenated preprocessed rs‐fMRI data of each group were subjected to ICA, utilizing Group ICA of fMRI Toolbox (GIFT) software (v4.0; TReNDS, https://trendscenter.org/software/). Independent components (ICs) were estimated at 37 (RHZ group) and 41 (HC group) by minimum description length (MDL) criteria, identified via Infomax algorithm.^32^, ^33^, ^34^ For each IC, the time courses mirrored waveforms of specific coherent brain activity patterns, and pattern intensities across voxels were expressed in corresponding spatial maps. To display voxels appropriate for individual ICs, intensities of each map were converted to z‐values.^35^ The DMN component of each group was extracted successfully, adopting a higher signal‐to‐noise ratio than that traditionally implemented.^36^, ^37^, ^38^ Subject‐specific spatial maps and time courses were then configured by temporospatial multiple regression back‐reconstruction approach in GIFT.

### 2.6 Second‐level analysis of the DMN

One‐sample *t* test was invoked for DMN spatial maps to evaluate within‐group data integrity (*P* < .05, false discovery rate [FDR] criterion corrected), powered by Statistical Parametric Mapping (SPM12). To further restrict DMN comparisons between groups, the DMN spatial maps of both groups were combined, creating a DMN mask. Two‐sample *t* test was engaged to determine between‐group differences in DMN spatial maps, with significance set at *P* < .05 (corrected by FDR and cluster size >43).

### 2.7 Seed‐based within‐DMN functional connectivity (FC) analysis

To examine the within‐DMN functional connectome, regions of interests (ROI) were extracted from significantly discrepant DMN spatial maps of RHZ and HC groups, based on two‐sample testing of above‐mentioned areas (including MFG, right/left MTC, right parietal lobe, right/left parahippocampal gyrus, and inferior frontal cortex [IFC]). We then identified one of these seven regions as ROI, performing voxel‐wise within‐DMN FC analysis by using the combined DMN spatial map as a mask. Correlation coefficients were ultimately converted (Fisher's z‐transformation) to a normal distribution. A total of seven voxel‐wise within‐DMN connectivity analyses were completed.

### 2.8 Statistical analysis

Demographic and clinical variables of RHZ and HC groups were assessed, expressing continuous variables as mean ± SD and testing for normality by Shapiro‐Wilk test. Intergroup differences in variables with no evidence against normality were subjected to independent two‐sample *t* test, applying chi‐squared or Fisher's exact test to categorical variables. All computations were driven by standard software (SPSS v24.0; IBM Corp), setting significance at *P* < .05.

Intergroup FC differences were assessed by two‐sided unpaired *t* test. To exclude possible confounding effects, age, sex, and education level were included as covariates in two‐sample testing.

When investigating correlations between abnormal within‐DMN FCs and clinical variables (duration, MPQ, VAS, PPI, ID Pain, HAMD, HAMA, PANAS, and SF‐36 scores), partial analyses were conducted, eliminating effects of age and education (in years), *P*‐values <.05 considered statistically significant. Because some pain‐related clinical variables (duration, MPQ, VAS, PPI, and ID Pain) were unavailable in the HC group, these were analyzed in the RHZ group only. All other clinical variables (HAMD, HAMA, PANAS, and SF‐36 scores) were available for partial correlation analyses of the combined groups.

## 3 RESULTS

### 3.1 Demographics and clinical characteristics

A total of 60 participants (RHZ group, 30; HC group, 30) were selected for this study. As shown in Table 1, there were no statistically significant differences between groups in terms of gender (RHZ: males/females, 16/14; HC: males/females, 14/16; *P* = .797) or age (RHZ: 61.63 ± 6.32 years; HC: 59.07 ± 7.47 years; *P* = .156), although duration of education differed significantly (RHZ: 9.20 ± 3.24 years; HC: 5.73 ± 4.41 years; *P* = .001). We also found that slight persistent pain existed in RHZ group members, as shown by VAS, MPQ (sensory/affective), PPI, and ID Pain scores, whereas pain‐related scores in the HC group were unavailable. Compared with the HC group, significantly higher HAMD (*P* = .006) and HAMA (*P* = .016) scores were observed in the RHZ group, while PANAS positive (*P* < .001) and life quality (SF‐36) scores (*P* < .001) were significantly lower. PANAS negative scores did not differ significantly by group (*P* = .689).

**Table 1:** Demographic and clinical variables of recuperative patients of herpes zoster (RHZ) patients and healthy controls (HCs)

	RHZ (n = 30)	HC (n = 30)	Statistics (*P*‐value)
Demographic data			
Age (y, mean ± SD)	61.63 ± 6.32	59.07 ± 7.47	.156
Gender (males/females)	14/16	16/14	.797
Education (y, mean ± SD)	9.20 ± 3.24	5.73 ± 4.41	.001*
Duration from first onset (mo, mean ± SD)	8.92 ± 5.62	0	
Clinical data			
Pain VAS(0‐10)	0.90 ± 0.99	0	
MPQ sensory	0.67 ± 0.76	0	
MPQ affective	0.13 ± 0.35	0	
PPI	0.53 ± 0.51	0	
ID Pain	0.30 ± 0.47	0	
Psychological data			
HAMD	2.67 ± 2.51	1.10 ± 1.58	.006*
HAMA	1.57 ± 1.57	0.67 ± 1.21	.016*
PANAS positive	15.00 ± 2.49	24.23 ± 2.31	＜.001*
PANAS negative	10.70 ± 1.37	10.57 ± 1.19	.689
SF‐36	127.56 ± 5.85	136.3 ± 3.0	＜.001*

Note

Two‐sample two‐tailed *t* tests were used for age, years of education, and psychological data comparisons between RHZ patients and HCs.

The chi‐squared test was performed for gender comparison between RHZ patients and HCs.

Abbreviations: HAMD, Hamilton Depression Scale; HAMA, Hamilton Anxiety Scale; HCs, healthy controls; MPQ, McGill pain questionnaire; PANAS, Positive Affect Negative Affect Score; PPI, present pain intensity; RHZ, recuperative patients of herpes zoster; SF‐36, 36‐item short form from health survey; VAS, visual analogous scale.

### 3.2 Spatial patterns of DMN

**Figure 1:**
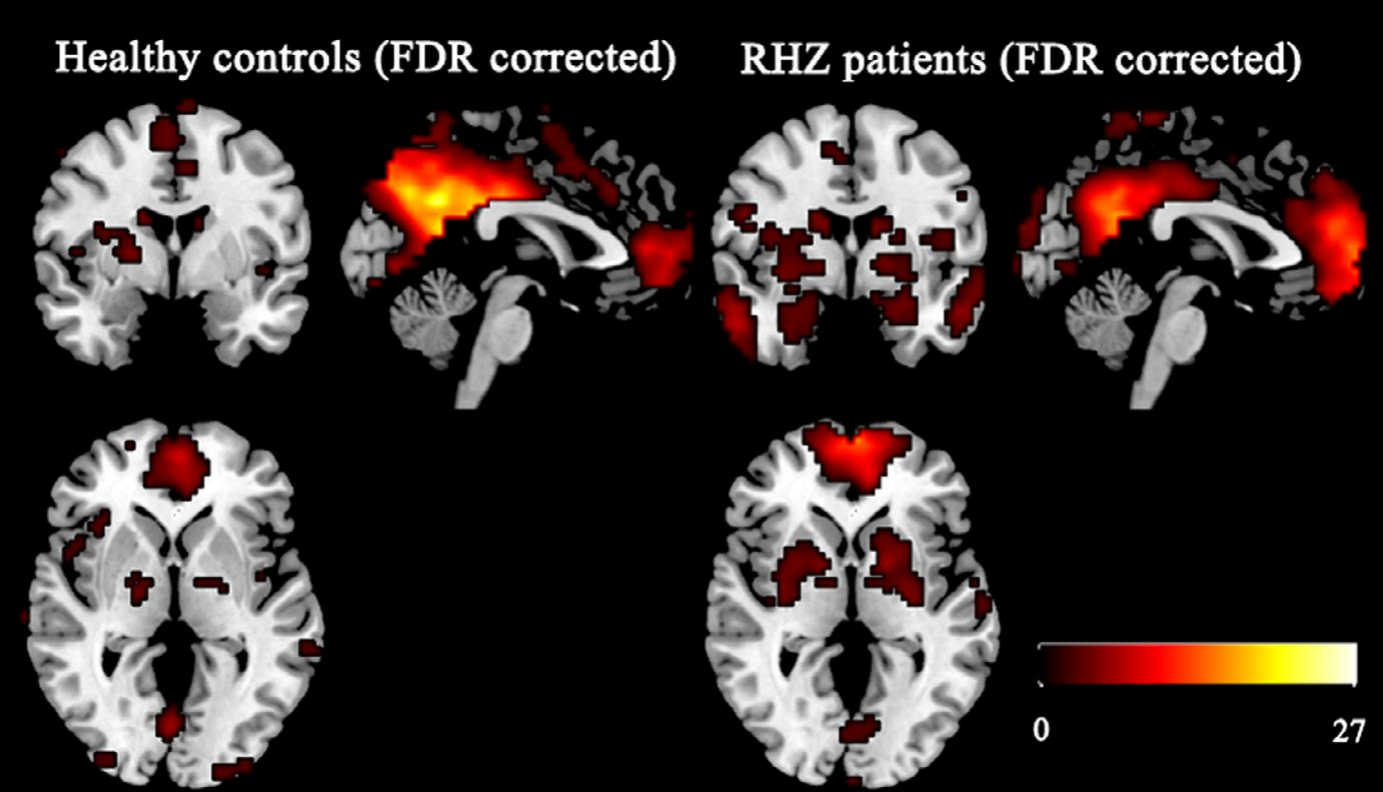
Patterns of DMN in RHZ patients and healthy controls (FDR corrected, *P < *.05). DMN, default mode network; RHZ, recuperative patients of herpes zoster

Among all components examined, the component co‐activating best with the DMN template was identified as each DMN pattern in respective group. To visualize group‐based DMN spatial maps, all group member components were pooled in a random‐effect analysis model (one‐sample *t* test) corrected by FDR (*P* < .05). Similarities in DMN patterns were thus demonstrated in two groups within the following structural areas: MFG, posterior cingulate cortex (PCC)/PCu, right/left MTC, parietal lobe, and hippocampus/parahippocampus (Figure 1).

### 3.3 Group differences in DMN

**Figure 2:**
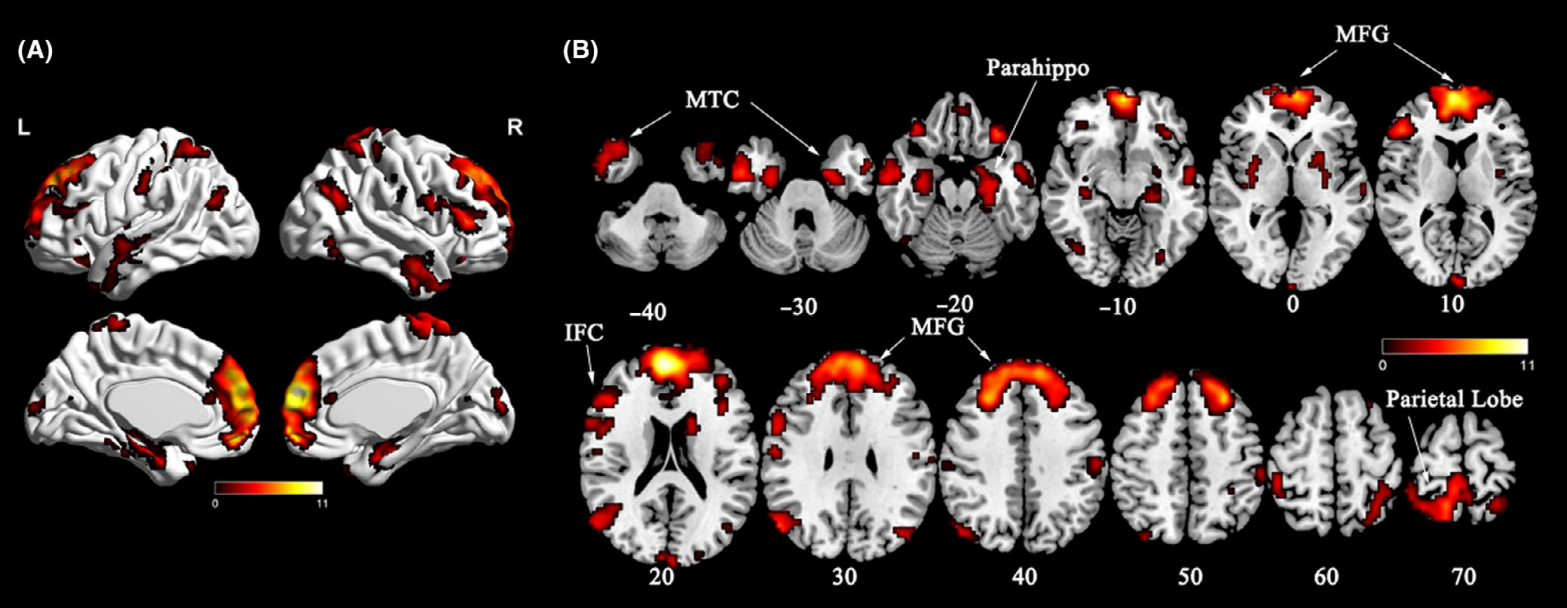
Voxel‐wised spatial map (SM) comparisons of DMN between HCs and RHZ patients. A, Compared to controls, RHZ patients displayed increased connectivity in the DMN regions, including MFG, right and left MTC, right parietal lobe, parahippocampal gyrus, and right inferior frontal cortex (FDR corrected, *P < *.05). B, Representative multislices of the discrepant spatial map of DMN between RHZ patients and HCs. DMN, default mode network; HCs, healthy controls; IFC, inferior frontal cortex; MFG, middle frontal gyrus; MTC, medial temporal cortex; Parahippo, parahippocampal gyrus; RHZ, recuperative patients of herpes zoster

In comparing DMN spatial maps by group, stronger connectivity within the DMN was shown for RHZ (vs HC) group, including MFG, right/left MTC, right parietal lobe, right/left parahippocampus, and IFC areas (*P* < .05, FDR corrected) (Figure 2A, Table 2). Representative multislices of discrepant DMN spatial map between two groups are provided in Figure 2B.

**Table 2:** Differences of DMN regions among RHZ patients and HCs. Comparisons were performed at *P < *.05 (FDR corrected)

Brain regions	L/R/B	Cluster size	Peak MNI coordinate			Peak T value
			X	Y	Z	
MFG	B	791	6	54	21	11.0
MTC	L	122	−57	−3	−6	3.82
	R	108	54	−3	−30	5.41
Parahippocampal gyrus	L	171	−27	−12	−24	5.32
	R	92	36	−21	−6	4.29
Parietal lobe	R	204	12	−54	66	6.52
Inferior frontal cortex	R	232	48	33	12	6.07

Abbreviations: B, Bilateral; L, Left; HCs, healthy controls; MFG, middle frontal gyrus; MNI, Montreal Neurological Institute; MTC, medial temporal cortex; R, Right; RHZ, recuperative patients of herpes zoster; X, Y, Z, coordinates of primary peak locations in the MNI space.

**Figure 3:**
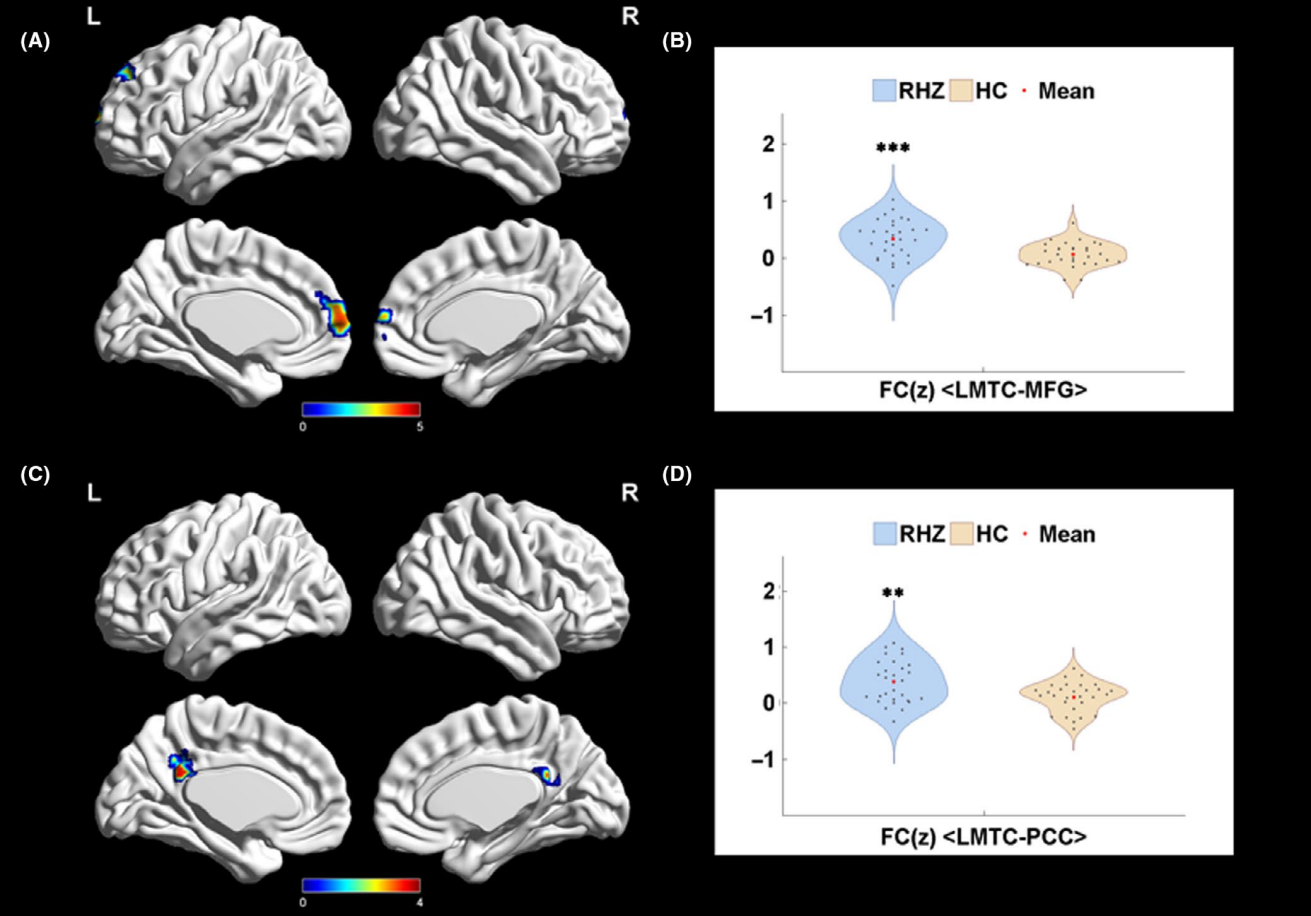
LMTC voxel‐wised within‐DMN connectivity. A, LMTC in RHZ patients displayed significantly increased connectivity to MFG (including mPFC) within DMN. B, The violin plots of LMTC‐MFG connectivity comparison between HCs and RHZ patients. C, LMTC in RHZ patients displayed significantly increased connectivity to PCC within DMN. D, The violin plots of LMTC‐PCC connectivity comparison between HC and RHZ patients. HCs, healthy controls; LMTC, left medial temporal cortex; MFG, middle frontal gyrus; PCC, posterior cingulate cortex; RHZ, recuperative patients of herpes zoster

Compared with the HC group, we found significant augmentation of LMTC‐MFG (including mPFC) connectivity (Figure 3A,B) and LMTC‐PCC connectivity (Figure 3C,D) of RHZ group. There were no significant between‐group differences in other ROI‐based voxel‐wise within‐DMN FCs, including ROIs of MFG, right MTC, parahippocampal gyrus, parietal lobe, and IFC.

### 3.4 Correlations between brain signals and clinical data

Because certain pain‐related clinical variables (duration, MPQ, VAS, PPI, ID Pain) were unobtainable in healthy controls, a partial analysis was undertaken, limited in the RHZ group, examining relations between these parameters and abnormal levels of within‐DMN FC. None of these variables correlated significantly with LMTC‐MFG or LMTC‐PCC connectivity in RHZ patients.

**Figure 4:**
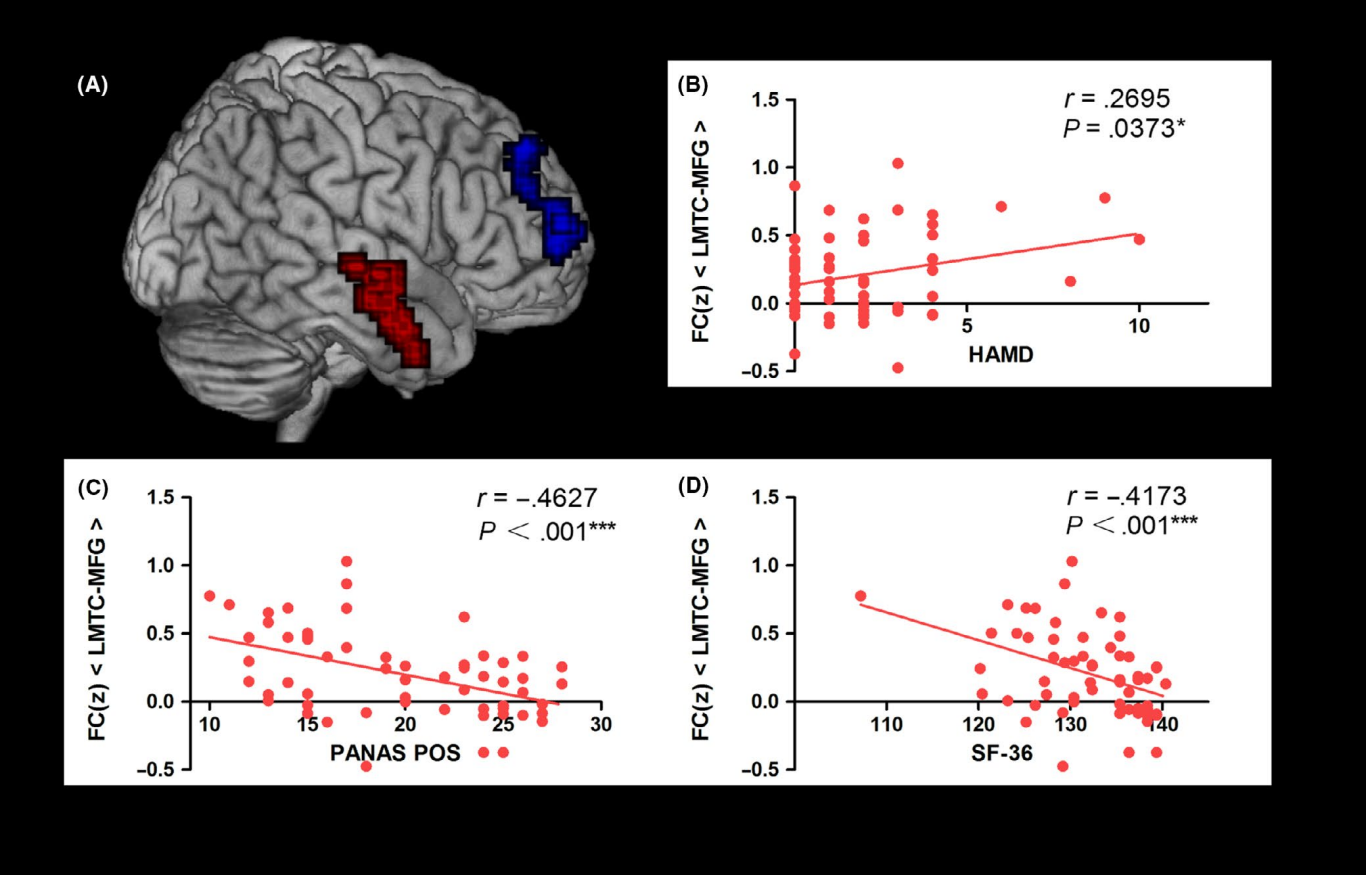
Correlation analysis between the LMTC‐MFG connectivity and clinical parameters. A, Locations in Montreal Neurological Institute space in the LMTC and MFG. Red, LMTC; Blue, MFG. B, The mean z value of LMTC‐MFG FC positively correlated with HAMD (*r* = .2695, *P* = .0373). C, The mean z value of LMTC‐MFG FC negatively correlated with PANAS positive scores (*r* = −.4627, *P < *.001). D, The mean z value of LMTC‐MFG FC negatively correlated with SF‐36 scores (*r* = −.4173, *P* < .001). HCs, healthy controls; LMTC, left medial temporal cortex; MFG, middle frontal gyrus; RHZ, recuperative patients of herpes zoster

Other clinical variables (HAMD, HAMA, PANAS, and SF‐36 scores) were available in both groups, so partial analyses proceeded in the combined groups. We subsequently discovered that levels of LMTC‐MFG FC correlated significantly with emotional alterations and life quality, showing a positive relation with HAMD (*r* = .2695; *P* = .0373) scores (Figure 4B) and negative relations with both PANAS positive (*r* = −.4627; *P < *.001) (Figure 4C) and SF‐36 (*r* = −.4173; *P < *.001) scores (Figure 4D). No significant correlations between intensity of LMTC‐MFG FC and PANAS negative (*r* = .0849; *P* = .519) or HAMA (*r* = .1745; *P = *.1824) scores were evident, nor did the intensity of LMTC‐PCC FC correlate significantly with clinical scores.

## 4 DISCUSSION

In this preliminary study, the following were major findings: (a) consistent with our initial hypothesis, DMN pattern disruption shown in the RHZ group (via ICA algorithm) indicated that patients recuperating from HZ deviate from the brain dynamics of healthy individuals, even after the pain of acute shingles resolves; (b) compared with healthy controls, levels of LMTC‐MFG and LMTC‐PCC connectivity are increased in the aftermath of zoster episodes; and (c) the intensity of aberrant LMTC‐MFG connectivity correlates with pain‐induced emotional alterations and life quality.

It is important to note that our study of the DMN was prompted by the existing body of knowledge on its role in a wide range of sensory and cognitive processing functions, including pain‐related rumination, attention, and memory.^16^, ^18^, ^39^, ^40^ Disruption of the DMN has been identified in many mental and psychological conditions, including Alzheimer's disease, traumatic brain injury, epilepsy, autism, and major depressive disorders.^41^, ^42^, ^43^, ^44^, ^45^ It is noteworthy that various neuroimaging studies have demonstrated disrupted DMN dynamics in conjunction with acute noxious pain (transitory stimuli in healthy subjects) and states of chronic pain due to migraine, chronic low back pain, or fibromyalgia.^21^, ^23^, ^24^, ^25^, ^26^ Our efforts are the first to delineate disrupted DMN patterns and altered levels of within‐DMN connectivity (LMTC‐MFG and LMTC‐PCC) in the recuperative phase of HZ. Hence, earlier reported DMN disturbances during acute HZ periods seem to be prolonged phenomena that endure after the characteristic pain is near‐extinct.^6^, ^7^, ^28^

**Figure 5:**
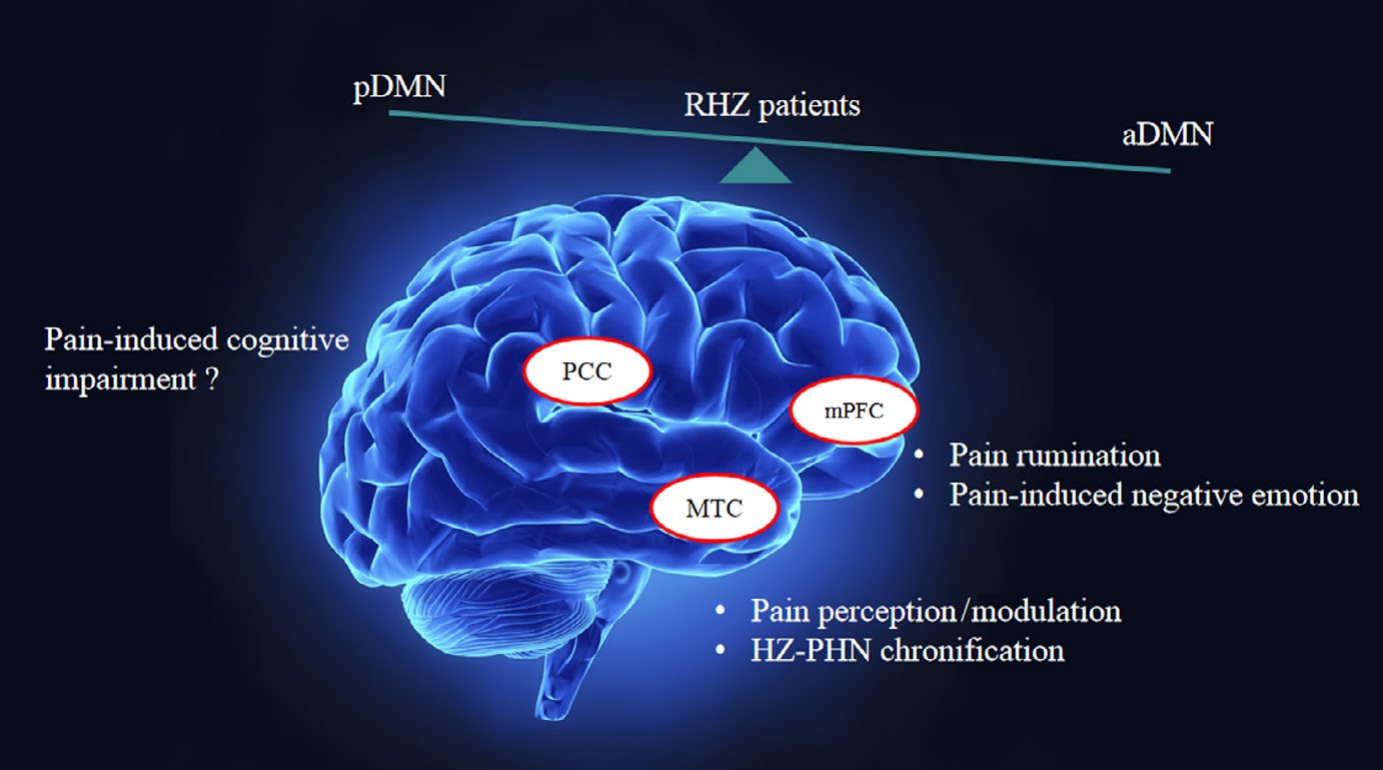
Schematic diagram of regions with aberrant FC within‐DMN and its related function in RHZ patients. The MTC may be related to pain perception, modulation, and HZ‐PHN chronification. The alternative LMTC FC within DMN in RHZ patients can be accounted for by the alteration of brain activity from acute herpes zoster to the recuperative period. The mPFC may be related to pain rumination and pain‐induced negative emotion and its dynamic alteration within‐DMN may not disappear in the short term after acute pain period. The PCC may be related to cognitive impairment, but whether RHZ patients show cognitive impairment, as seen in PHN patients, warrants further investigation. In summary, RHZ patients showed disequilibrium in anterior and posterior subnetworks after acute herpes zoster pain. HCs, healthy controls; MFG, middle frontal gyrus; MTC, medial temporal cortex; PCC, posterior cingulate cortex; RHZ, recuperative patients of herpes zoster

The three aforementioned DMN regions (LMTC, MFG, and PCC) marked by abnormal connectivity are key nodes in pain regulation. Temporal cortex is one area of the brain involved in pain perception and modulation, as confirmed in human neuropathic states and in animal models.^46^, ^47^, ^48^ Past studies have demonstrated that abnormal LMTC function and structure are associated with the spontaneous pain and allodynia of HZ or PHN.^7^, ^8^, ^10^, ^49^ In the chronification process transitioning from acute HZ to PHN, functional and pain‐duration‐dependent gray matter volume (GMV) changes are shown in the temporal gyrus,^7^, ^50^ an area critical in pain generation and chronification. The heightened LMTC seed‐based connectivity we observed in the RHZ group may well represent residual abnormal connectivity from prior periods of acute pain propagation, despite near‐absence of clinically apparent pain (Figure 5).

The mPFC is a key component of MFG, exerting dual and opposing roles in pain.^51^ Antinociception may be conveyed through its connections with other cortical areas and the descending pain modulatory system, most notably in the periaqueductal gray (PAG).^52^, ^53^ On the other hand, the chronification of pain may be promoted through corticostriatal projections, perhaps relying on dopamine receptor activation (or lack thereof) in the ventral tegmental area‐nucleus accumbens (VTA‐NAc) reward pathway.^54^, ^55^, ^56^ There is also neuroimaging evidence that patients with chronic pain display enhanced connectivity between mPFC and other DMN regions, such as PCC/PCu and retrosplenial cortex, linked to pain catastrophizing and rumination (ie, a form of thought characterized by repetitive attention to discomforting pain stimuli and negative emotions).^57^, ^58^ Those afflicted with chronic pain also bear abnormal interactions between DMN and the descending modulatory system, viewed as an underlying mechanism of rumination on chronic pain.^22^ Moreover, difficult control of negative thinking that accompanies abnormal DMN activity may culminate in emotional dysregulation and aggravate depressive behavior in adolescents with mood disorders.^59^, ^60^, ^61^ A vicious cycle is triggered by pain and depression, based on overlap of structures (ie, mPFC, insula, NAc, and amygdala), circuits, receptors, and neurotransmitters.^62^, ^63^, ^64^ The connectivity of mPFC with other DMN regions and with other pain‐related areas of the brain is thus a factor in pain rumination and pain‐induced depression and may explain the relation we established between mPFC seed‐based within‐DMN connectivity and pain‐induced emotional alteration in the RHZ group. Although our recuperating population did not transition to PHN, they experienced several weeks of protracted HZ pain, along with rumination‐related within‐DMN connectivity alterations, and the changes were not short term in nature (Figure 5). We speculated these connectivity abnormalities were remnants of acute HZ.

A recent study has further shown that patients with chronic pain due to spondyloarthritis exhibit complex relations between pain, resilience, and mPFC seed‐based within‐DMN connectivity. Resilience is a positive psychological factor, enabling rebound in response to adverse events, like pain.^65^, ^66^ In the setting of chronic pain, resilience is linked to pain catastrophizing, adjustment, and acceptance through positive emotional experiences and may be predictive of pain‐related health outcomes.^65^, ^67^, ^68^ A link between mPFC‐related within‐DMN connectivity and resilience is thus presumed in patients recuperating from RHZ. In other words, DMN‐related resilience may invigorate the recovery process and boost rates of recuperative transformation by increasing positive emotions.

As a pivotal subregion of the mPFC, ventral medial prefrontal cortex (vmPFC) is often considered a sensory‐visceromotor aggregator associated with mood regulation, motivation, and social behavior.^69^ Neuroimaging studies indicate that individual emotional states directly affect vmPFC activity.^19^ The diminished vmPFC activity negatively correlates with anxiety self‐ratings, reflecting a dynamic balance between attention and certain anxiety states.^70^, ^71^ In our patients, there was a trend toward positive correlation between mPFC‐related within‐DMN connectivity and anxiety scores in the RHZ group, falling short of statistical significance. Nevertheless, this raises the possibility that increased mPFC‐related connectivity in patients recuperating from HZ may signal disruptions in anxiety states and individual brain homeostasis (Figure 5).

Posterior cingulate cortex, another key node of DMN, is activated during attention regulation and is extensively connected to medial temporal lobe memory systems.^72^, ^73^ However, there have been no consistent conclusions regarding PCC connectivity with other DMN regions in instances of acute or chronic pain. Diminished PCC connectivity within the DMN has been reported in patients with chronic back pain, complex regional pain syndrome, osteoarthritis, and tonic pain stimuli; and the intensity of PCC connectivity seems to increase in those with temporomandibular disorders.^57^, ^74^ Thus, the increased LMTC‐PCC connectivity observed in our RHZ group members may underlie an altered ability for some particular tasks, such as attention and memory (Figure 5). Whether they also may develop cognitive impairment (as in patients with PHN) is the subject of further investigation.^75^

Finally, recent studies have contended that anterior (mPFC) and posterior (PCC) DMN subnetworks achieve a dynamic balance vital for maintenance of cognitive function.^76^ In our study, the aberrant anterior and posterior within‐DMN connectivity shown by the RHZ group seems to link such zonal disequilibrium with pain‐induced rumination and emotional regulation after extinction of acute protracted pain. This finding enhances our understanding of reciprocity between subnetworks responsible for sensory, emotional, and cognitive dimensions of pain (Figure 5).

The current preliminary study has limitations that may be remedied by future endeavors. Insufficient data acquisition in patients with acute HZ or PHN prevented analysis of DMN differences in acute, recuperative, and persistent (PHN) phases of HZ, an issue addressed by cross‐sectional and longitudinal imaging studies currently underway. Longitudinal imaging investigations are needed to identify the evolution of DMN alterations during episodes of HZ. Other large‐scale networks and their internal/external connectivity implications should also be considered and are presently being explored. Finally, we did not measure behavioral changes through cognitive or attention‐demanding tasks or questionnaires to prove a correlation between intensity of PCC‐related connectivity and cognitive performance.

In conclusion, the present efforts are the first to characterize disruption of DMN patterns and within‐DMN connectivity in patients recuperating from HZ, despite resolution of major symptoms (ie, rash and pain). We have also determined a relation between within‐DMN connectivity and pain‐induced negative emotional states, attributing residual pain rumination, emotional alterations, and resilience to DMN divergence. Our preliminary results may help clarify the brain dynamics during recovery that likely persist well beyond the acute period, providing heuristic cues for additional research on neuromechanisms of HZ transition.

## CONFLICTS OF INTERESTS

The authors declare no conflict of interest.
